# Callers’ Descriptions of Stroke Symptoms during Emergency Calls in Victims Who Have Fallen or Been Found Lying Down: A Qualitative Content Analysis

**DOI:** 10.3390/healthcare12040497

**Published:** 2024-02-19

**Authors:** Veronica Lindström, Mihaela Oana Romanitan, Annika Berglund, Ruxandra Angela Pirvulescu, Mia von Euler, Katarina Bohm

**Affiliations:** 1 Department of Nursing, Umeå University, 90187 Umeå, Sweden; veronica.lindstrom@umu.se; 2 Department of Health Promotion, Sophiahemmet University, 11486 Stockholm, Sweden; 3 The Ambulance Service, Region of Västerbotten, 90189 Umeå, Sweden; 4 Department of Internal Medicine, Södersjukhuset, 11883 Stockholm, Sweden; mihaela.romanitan@ki.se; 5 Karolinska Institute’s Stroke Research Network at Södersjukhuset, Department of Clinical Science and Education, 11883 Stockholm, Sweden; katarina.bohm@ki.se; 6 Department of Clinical Neuroscience, Karolinska Institutet, 17177 Solna, Sweden; annika.berglund@regionstockholm.se; 7 Ophthalmology Department, University of Medicine and Pharmacy “Carol Davila”, 020021 Bucharest, Romania; 8 Department of Neurology, Örebro University Hospital, 70185 Örebro, Sweden; mia.von-euler@oru.se; 9 School of Medical Sciences, Örebro University, 70182 Örebro, Sweden; 10 Department of Emergency Medicine, Södersjukhuset, 11883 Stockholm, Sweden

**Keywords:** dispatch centre, emergency call, stroke, content analysis

## Abstract

Early identification of stroke symptoms is essential. The rate of stroke identification by call-takers at emergency medical communication centres (EMCCs) varies, and patients who are found in a lying down position are often not identified as having an ongoing stroke. Objectives: this study aimed to explore signs and symptoms of stroke in patients who had fallen or were found in a lying position. Design: a retrospective exploratory qualitative study design was used. Method: a total of 29 emergency calls to EMCCs regarding patients discharged with a stroke diagnosis from a large teaching hospital in Stockholm, Sweden, in January–June 2011, were analysed using qualitative content analysis. Results: during the emergency calls, the callers described a sudden change in the patient’s health status including signs such as the patient’s loss of bodily control, the patient’s perception of a change in sensory perception, and the callers’ inability to communicate with the patient. Conclusions: The callers’ descriptions of stroke in a person found in a lying position are not always as described in assessment protocols describing the onset of a stroke. Instead, the symptom descriptions are much vaguer. Therefore, to increase identification of stroke during emergency calls, there is a need for an increased understanding of how callers describe stroke symptoms and communicate with the call-takers.

## 1. Introduction

Stroke, one of the causes of mortality and disability worldwide, is a time-dependent medical emergency. Reperfusion treatment is effective only if provided within a specific time frame, so early presentation at the hospital is important [1,2]. Not identifying stroke during an emergency call can cause a delay in treatment [3]. Early identification of stroke is essential to enable effective reperfusion treatment [2]. However, too few patients arrive at the emergency departments within the time frame for administration of thrombolysis treatment, and a delayed identification of stroke during the emergency call can be part of the cause [4]. Therefore, one way to improve the chance of reaching the point of care within the specific time frame is to identify patients experiencing a stroke during the emergency call. Identifying patients experiencing a stroke based on a phone call is difficult, particularly if the caller is a third person who was not present during stroke onset. Previous studies have shown that the rate of stroke identification by call-takers at emergency medical communication centres (EMCCs) varied from 31% to 57% [5,6,7,8,9,10,11,12]. The challenges in assessing emergency calls and identifying stroke may arise because the EMCC personnel seldom have extensive stroke case experience, the caller is often a third person, and the patient cannot be seen or examined [5,13,14]. Additionally, it has been previously shown that patients who were discovered in a lying down position were frequently not identified as experiencing an ongoing stroke during the emergency call. Early identification of stroke symptoms is essential and exploring the callers’ descriptions of stroke symptoms may clarify which signs and symptoms reveal a stroke. This understanding can make it easier for call-takers at EMCCs to identify an ongoing stroke. This study aimed to explore signs and symptoms of stroke in patients who had fallen or were found in a lying position.

## 2. Materials and Methods

### 2.1. Design and Setting

A retrospective exploratory qualitative study design was used. This is a sub-study of a larger study carried out in a major teaching hospital in the city of Stockholm, Sweden in 2011. Patients transported by ambulance to the hospital and discharged with stroke diagnosis were included in the study after informed consent was obtained. A total of 179 patients were included in the study [15]. During the study period, 18 EMCCs were involved; the majority (95%) were managed by the public-owned company SOS Alarm. The EMCCs handled all the 112-emergency calls and were responsible for dispatching the ambulances. During the study period, a call to an EMCC was initially answered by a first call-taker without formal medical training, and after establishing the patient’s level of consciousness and patterns of breathing and collecting the patient’s personal data, the call was forwarded to a registered nurse (RN). The RN assessed the patient’s condition and the level of acuity and forwarded information regarding priority to a dispatcher. To support the assessment, the call-taker and the RN used an electronic protocol, the Swedish Medical Index, which consists of 34 main units [16] which are mainly based on symptoms and a series of questions to ask the caller. There are four levels of acuity in the assessment protocol: priority 1—acute life-threatening situation/condition; priority 2—acute but not life-threatening; priority 3—transportation to health care facility required; priority 4—no medical aid needed during transport. Unlike priorities 1–3, priority 4 is not regulated by the Swedish board of health. If the stroke is suspected to have occurred within six hours of the call, the case is given the highest dispatch priority, and an ambulance is dispatched during the call. The Standard for Reporting Qualitative Research (SRQR) has informed the reporting of the current study [17].

### 2.2. Reporting Patient and Public Involvement

With informed consent, the patients were asked about participation in the study and informed that analysis of their emergency calls would be made. The patients were not involved in the study’s design nor in disseminating information about the study. The study was retrospective and did not require any extra time from the participants.

### 2.3. Data Collection

Purposeful sampling was used for data collection [18]. The purposefulness was ensured by including emergency calls that described patients experiencing an acute stroke and had fallen or were found in a lying down position. Out of 179 emergency calls analysed by Berglund et al., 38% (*n* = 68) of the patients experienced falling or were found in a lying down position by the caller. In this study, 29 of the 68 emergency calls were available for further analysis. The same sub-set was used in another qualitative study using interpretive phenomenology [19] when analysing the data. The included calls were deemed sufficient and manageable for qualitative content analysis to explore the callers’ descriptions of stroke symptoms of patients who experienced falling or were found in a lying down position. The included emergency calls were transcribed verbatim. No information about the caller was collected other than what was collected during the call. The caller was a neighbour in six calls, a professional without formal medical education (i.e., police, homecare provider) in nine calls, and in two calls the patient him/herself was the caller. No additional information regarding patient or caller characteristics was collected from the calls, as the calls did not contain that type of information.

### 2.4. Analysis

The transcribed text was analysed using qualitative content analysis [18] to explore the callers’ description of symptoms when the care seeker experienced an acute stroke and was found in the lying down position. The first part of the analysis consisted of reading the text several times to gain an understanding of the content of the emergency call. Next, the text was divided into meaningful units, i.e., words, phrases, and/or sentences related to each other by the content and the explored phenomenon, the onset of stroke. The meaningful units were then summarised into condensed meaningful units, i.e., descriptions close to the transcriptions of the emergency call [18]. The descriptions were then compared, discussed, and interpreted by the authors to achieve a consensus on their meaning. During this part of the analysis, four sub-themes were extracted. During the process of analysis, there was constant movement forward and backward between the parts of meaningful units, condensed meaningful units, and sub-themes. The aim was to preserve the core of the callers’ descriptions of stroke in patients found in the lying position. Finally, after several discussions among the authors, a consensus was reached and out of the four sub-themes, one main theme was formulated.

### 2.5. Ethical Considerations

The study complied with the ethical principles of research described by the International Council of Nurses [20], which requires researchers to ensure anonymity, integrity, and confidentiality of the callers, patients, call-takers, and EMCCs. Informed consent was obtained from the patients concerned in accordance with the ethical permit from the Regional Ethics Board (EPN 2010/703-31/2, CPN Ö 13-2010, and additional approval 2012/2055-32). Callers as well as call-takers were anonymous and not identified, and in accordance with the ethical approval no consent was requested.

## 3. Results

**Figure 1:**
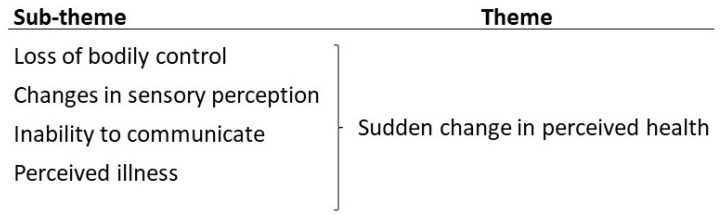
The four sub-themes and one theme describing the callers’ description of stroke in patients found lying down.

Four sub-themes and one theme were identified from the analysis, as displayed in Figure 1.

### 3.1. Sudden Change in Perceived Health

During the emergency call, the callers described a sudden change in health status as a loss of bodily control, changes in sensory perception, inability to communicate with the patient, and perceived illness. The callers also described the change in health with a time frame and compared it with the patient’s previously normal health status.

### 3.2. Loss of Bodily Control

In the sub-theme ‘loss of bodily control’, there were several different descriptions of the body’s position, the patient’s ability to move, and how the patient had lost control over his/her body: ‘…*he fell from the toilet…*’, ‘…*his body is not upright…*’, ‘…*she just lies there…she is not able to move her body…*’, ‘…*my brain is clear but my leg cannot carry me…*’, ‘… *the eyes are rolling…*’, and ‘…*he lies there… just looking up and into a corner*...’. There were also descriptions of stroke in accordance with how the medical literature describes hemiparesis and facial paresis, e.g., an arm and/or a leg limb did not function, and the face looked skewed.

The callers also talked about the presence of seizures (with or without micturition) in some of the calls. Despite the differences in the descriptions given in the emergency calls, all the callers said that the patient had lost control over their body to some extent.

### 3.3. Changes in Sensory Perception

The sub-theme that surfaced in the description of changes in the patient’s sensory perception was the description of pain located somewhere in the body, headache, dizziness, numbness in a limb, hearing loss, or loss of sensations. There were also emotional changes in the patient described by the caller, such as ‘*… she is so sad…*’ or ‘*she is crying*’. The symptoms describing changes in the sensory perception were presented as a single sensation, e.g., ‘…*she has a terrible headache…*’, and as a combination of sensations, e.g., ‘*I suddenly got a terrible sound in one ear*, *and then I became dizzy…*’ and ‘…*it hurts in the head and in the arms*’.

### 3.4. Inability to Communicate

Another sub-theme was the patient’s inability to communicate, usually explained with the phrases ‘…*cannot speak*’, ‘*the patient slurs…*’, and ‘…*he*’*s not saying anything*’. The ability to communicate was also affected by the patient’s alertness. When the patient was described as not being awake, the communication was limited: ‘*He is completely powerless*, *and it is difficult for him to talk to*...’. The ability to communicate was also affected when the patient suffered memory loss, e.g., ‘*…he seems not to remember anything…*’, and when the patient did not understand or could not follow the given instructions. When a third person described the patient’s inability to communicate, he/she mostly described the symptoms as a practical dysfunction, e.g., inability to speak, understand, or respond.

### 3.5. Perceived Illness

The sub-theme of perceived illness included descriptions of the perception of the patient’s general health status and the caller’s (or patient’s) own suspicion of what the patient was experiencing. When describing the perceived illness or suspected disease, the callers used descriptions such as ‘*he*’*s a bit warm…*’, ‘*he*’*s really tired…*’, ‘*he*’*s not doing well…*’, ‘*something has happened…*’, and ‘*he looks very bad…*’. The callers also tried to explain the symptoms by giving their own diagnosis: ‘*I think I*’*m having a heart attack…*’ or ‘*it looks like a stroke…*’. The perceived illness was described by the patients, next of kin, and professionals without formal medical education.

## 4. Discussion

This study shows how callers describe stroke in patients found lying down and can serve as a basis to discuss how these descriptions may affect stroke identification and response. The study provides a better understanding of the leading causes for missing a stroke diagnosis during 112 calls, which can lead to a higher number of stroke patients admitted at emergency departments in the time frame for thrombolysis treatment and consequently may lead to better outcomes. A better understanding of the complexity of assessing emergency calls related to patients experiencing a stroke could lead to the proper medical education of 112 operators to identify stroke symptoms. The callers’ descriptions of stroke were not always concordant with how assessment protocols and information for the public and surrounding community describe signs and symptoms of stroke. Instead, the symptom descriptions were vague—the callers mention loss of bodily control, changes in sensory perception, inability to communicate, and perceived illness—which can lead to frequent missed stroke diagnoses. However, even though the callers’ descriptions of signs and symptoms may vary, the described symptoms are rather similar to stroke symptoms as described in information given to the general public on when to suspect a stroke and call the emergency number [21]. The Face-Arm-Speech-Time (FAST) test is commonly used in communicating stroke symptoms [22,23]. Although not specific, all the emerging themes correspond to FAST symptoms. The theme ‘perceived sudden change in health’ is a vague description but it does pinpoint the sudden onset (the time) of stroke. The theme ‘Loss of bodily control’ is related to the Arm part of the FAST test. And finally, ‘inability to communicate’ is directly reflected in the Speech part of FAST. The theme ‘perceived illness’ does not directly correspond to FAST. Except the Los Angeles Prehospital Stroke Scale, prehospital stroke identification tests include speech disturbance [22]. All prehospital stroke identification tests include arm weakness, and many also include leg weakness, i.e., findings that correspond to the theme ‘loss of bodily control’ [22]. However, it was apparent from the analysed emergency calls that the personnel assessing the emergency call did not identify the described symptoms as descriptions of a stroke. This may indicate that callers and the call-takers may have barriers in communication. It may also indicate that the call-takers lacked training, competence, and/or experience.

The issue of being unable to identify stroke may also be attributed to the complexity in the callers’ descriptions of stroke symptoms, as described by Prabhakaran et al. [14]. Regardless of the reasons behind call-takers not identifying stroke cases in the analysed emergency calls, the findings of our study reveal that understanding of how the callers communicate with the 112-emergency operators when describing a patient’s symptoms needs to be improved.

Our results are similar to those of a study involving patients with sepsis during emergency calls. Both stroke and sepsis are time-dependent medical emergencies, and treatment can be extremely effective if provided early [1,2,24]. This may indicate that instead of focusing on identification of diagnoses such as stroke or sepsis, the assessment protocol should support the personnel assessing the emergency call to identify time-dependent medical emergencies. However, further research is needed to verify or reject this assumption. Another way could be to develop follow-up questions for the call-takers such that the symptoms available for assessment are reduced.

In all the calls analysed in the study, there was a description of a sudden change in perceived health, and similar findings were described by Prabhakaran et al. [12]. A sudden change in perceived health in combination with other symptoms, such as a loss of bodily control, could be used as a trigger to identify stroke or a time-dependent medical emergency during the call, but further research is needed to verify what a sudden change in perceived health includes. Furthermore, there is a need to identify what time frames the callers are referring to when describing ‘a sudden’ change in health.

As previously shown, identifying stroke in patients found in the lying down position is difficult [5,14]. Our study’s findings indicate that in addition to communication barriers there may be other factors such as the influence of how the callers communicate with the call-takers on the assessment of the emergency call. It remains to be explored whether the availability of ambulances, the number of calls waiting to be answered, or other distracting factors may also be the reasons behind emergency call-takers not being able to identify patients experiencing a stroke.

### Strengths and Limitations

The results should be interpreted in the context of the following methodological limitations. Overall, the generalisability of the results is limited, but it is reasonable to think that the outcomes can be transferable to similar contexts since the analysed calls were concerned with stroke and were made by a variety of callers, patients, next of kin, and professionals without formal medical education. Another limitation may be that the calls analysed were made in 2011, but it is reasonable to assume that symptom presentation has not changed over time because the symptoms of stroke are the same as before. The purposeful data collection was intended to provide an insight into how the callers describe signs and symptoms in patients found lying down, and our findings show the complexity of assessing emergency calls. The callers described other aspects of stroke symptoms than those in the literature, but further research is needed to verify this finding. Furthermore, we did not have much knowledge about the callers; a better description of the callers could have enabled us to understand their descriptions of symptoms. To ensure credibility of the analysis, member checks were made continuously, starting with the selected meaning units. The aim of the member check was to avoid the use of very long or very narrow meaning units. Agreement among the authors was also sought during the analysis, which may not have increased the credibility [25], but it resulted in reflexive discussions about different perspectives among the co-researchers of descriptions of stroke in patients found lying down. The authors have different professional and scientific backgrounds, and this may have deepened the analysis.

## 5. Conclusions

The callers’ descriptions of stroke in a person found in a lying position are not always the same as those in assessment protocols regarding the onset of stroke. Instead, the symptom descriptions are vague, such as loss of bodily control, changes in sensory perception, inability to communicate, and perceived illness, which can easily make difficult the early identification of stroke symptoms, with the consequence of late arrival to the emergency department and missed thrombolysis treatment. Increasing the identification of stroke during emergency calls is crucial since the treatment of choice of acute thrombolysis within a specific time frame can be missed, with major consequences for the outcome as well as economic consequences with respect to longer hospitalization and a longer recovery for those patients.

## 6. Strengths and Limitations of This Study

A strength of the study is that it provides a picture of an authentic situation since the calls were analysed without the callers and the calltakers awarness of the study.

It is valuable to describe how patients with stroke who have fallen or been lying down are described in the emergency call as this has been identified in previous studies as a knowledge gap.

A limitation of the study is that patients with severe stroke and dysphasia were probably not included in the study due to problems giving informed consent and only 57% of eligible patients agreed to participate in the study.

## Data Availability

The meaning units and codes used for analysis during the current study are available from the corresponding author on request.

## Abbreviations

EMCC	Emergency medical communication centre
RN	Registered nurse
